# Research on the design and innovative transformation path of shoulder rehabilitation wall-climbing ladders for postoperative breast cancer patients based on design thinking

**DOI:** 10.1186/s12912-026-04689-7

**Published:** 2026-04-27

**Authors:** Wenhao He, Piao Chen, Chunhua Chen, Huiying Qin, Le Xia, Huiting Zhang

**Affiliations:** 1 Department of Breast Cancer, Sun Yat-sen University Cancer Center, 651 Dongfeng East Road, Guangzhou, Guangdong 510060 China; 2 State Key Laboratory of Oncology in South China, Guangzhou, China; 3 Guangdong Provincial Clinical Research Center for Cancer, Guangzhou, China; 4 Department of Nursing, Sun Yat-sen University Cancer Center, 651 Dongfeng East Road, Guangzhou, Guangdong 510060 China; 5 Kiang Wu Nursing College of Macau, Macao, China

**Keywords:** Design thinking, Breast cancer, Postoperative rehabilitation, Rehabilitation device, Nursing innovation

## Abstract

**Objective:**

To develop a shoulder rehabilitation wall-climbing ladder for postoperative breast cancer patients using a design thinking–driven approach, and to evaluate its usability, feasibility, and translational application in clinical and home settings.

**Methods:**

This study integrated a design thinking framework (Empathize–Define–Ideate–Prototype–Test) with real-world implementation. Clinical observations, literature review, and semi-structured interviews with patients and nurses were conducted to identify limitations of existing wall-climbing approaches and define core design requirements. Following iterative prototyping and optimisation, the final product was implemented across multiple hospitals. Patient satisfaction and nurse user experience were assessed using self-developed questionnaires.

**Results:**

The device was implemented in 148 hospitals, with 5,555 units distributed. A total of 199 patients reported a mean satisfaction score of 42.66 ± 5.30 (out of 50), with 86.0% indicating high satisfaction. Among 119 nurses, the mean user experience score was 47.88 ± 3.49, with 96.6% reporting high endorsement. Users highlighted ease of use, improved comfort, and facilitation of standardized rehabilitation and home-based exercise. Suggested improvements focused on enhancing quantitative assessment and recording functions.

**Conclusion:**

This study demonstrates a feasible nursing innovation pathway integrating design thinking with real-world implementation. The device shows good usability in both ward and home settings and may support standardized postoperative rehabilitation after breast cancer surgery. Further studies are needed to evaluate its clinical effectiveness.

**Supplementary Information:**

The online version contains supplementary material available at 10.1186/s12912-026-04689-7.

## Introduction

Breast cancer has become one of the most prevalent malignant tumours among women in China [1, 2]. With advances in comprehensive treatment, overall patient survival has markedly improved, making postoperative upper limb functional recovery and quality of life critical clinical and nursing priorities [3]. Radical mastectomy and axillary lymph node dissection may cause restricted shoulder joint mobility, pain, and adhesions on the affected side. Without standardized, sustained functional exercise, upper limb dysfunction severely impairs activities of daily living and may exacerbate negative emotions such as anxiety and depression [4–6]. “Wall-climbing” is a commonly employed method for active shoulder joint mobilization following breast cancer surgery. Its simplicity and lack of complex equipment requirements make it widely used in ward education and home rehabilitation [7–9]. However, current clinical practice and literature indicate three predominant approaches and associated issues: (1) Manual wall-climbing: Patients are instructed to select a wall in their ward or home for “finger wall-climbing”, with some marking graduations on the wall using chalk. This method requires no additional equipment, but walls are often smooth, causing noticeable friction between fingers (particularly fingernails) and the surface. In our clinical observations and patient feedback, some patients describe this sensation as “uncomfortable nail scraping against the wall”, which may reduce motivation for long-term adherence. Furthermore, most domestic walls lack standardized markings, making it difficult to quantify training height and progress. (2) Marked-sticker wall-climbing: Some literature reports affixing long, height-marked adhesive strips to walls, guiding patients to climb along these markers [10]. While this resolves the “no markings” issue, the smooth, flat surface of the stickers offers no fixed grip points for fingers, resulting in poor leverage and stability. Patients still rely on friction from fingertips for support, leading to discomfort and reduced safety. The stickers also tend to curl or peel off. (3) Large fixed apparatus wall-climbing systems: Certain large shoulder joint trainers currently available on the market, primarily intended for rehabilitation departments, are typically bulky and constructed from heavy materials, often requiring wall drilling for installation.While these devices offer relatively standardized training pathways, their high cost renders them unsuitable for widespread installation in standard breast surgery wards. They are also impractical for patients to purchase and install at home, limiting their universal adoption in breast surgery settings [11, 12]. Whether involving manual wall-climbing, affixing calibrated stickers to walls, or relying on large fixed rehabilitation apparatus, these approaches all exhibit varying degrees of discomfort, difficulty in quantifying training height, high equipment costs, and inconvenience for widespread deployment in standard wards or home settings [10–12]. These factors compromise patient long-term adherence and rehabilitation training efficacy.This situation indicates an urgent need for innovative nursing tools and service models that better align with clinical settings and facilitate widespread adoption in post-operative upper limb functional rehabilitation for breast cancer patients.

The nursing sector is encouraged to strengthen collaboration between research and clinical practice, promoting the translation and application of technological and device innovations. However, despite numerous nursing innovation patents in China, standardized pathways for translation and scalable implementation models remain underdeveloped, constraining the full realisation of nursing innovation’s value [13].

Design thinking is a human-centered systematic innovation methodology that emphasizes an iterative process of “empathy - problem definition - ideation - prototyping - testing”. This approach integrates multidisciplinary knowledge and stakeholder perspectives to identify product and service solutions better aligned with user needs within authentic usage contexts [14, 15]. During the empathy and definition phases, designers gain deep insights into users’ authentic contexts and pain points through observation and interviews, synthesizing fragmented experiences into clear problem statements. In the ideation and prototyping stages, diverse creative ideas are encouraged alongside rapid development of low-cost prototypes or minimum viable products for early validation in real-world scenarios. During the testing phase, designers continually refine or even redefine problems based on user feedback, thereby establishing a user-centered, iterative innovation process [15, 16]. In recent years, design thinking has progressively been applied within healthcare and nursing domains to guide medical device development, optimise health service processes, and enhance patient experiences. This approach facilitates greater alignment between nursing innovations and clinical practice, providing methodological support for subsequent translation and application [17–20].

Consequently, this study employs design thinking as its methodological framework. Centering on critical pain points in postoperative shoulder rehabilitation for breast cancer patients, a multidisciplinary team conducted a full-process practice encompassing empathy, problem definition, ideation, prototyping, and testing. The objective is to develop an integrated rehabilitation tool that facilitates both ward-based education and home rehabilitation without significantly increasing patients’ financial burden, thereby providing a reference pathway for the standardized translation and dissemination of nursing innovations.

## Methods

### Research design

This study integrates design research with real-world dissemination. A design thinking framework was first applied to explore user needs and define key limitations of existing wall-climbing exercise methods. Based on this process, a shoulder rehabilitation wall-climbing ladder was developed in terms of both structure and function, and subsequently granted a national design patent. The device was then implemented and promoted in breast surgery departments across multiple hospitals through the LOVE BREAST program. User evaluations from patients and nursing staff were collected using questionnaires.

This study was approved by the Ethics Committee of Sun Yat-sen University Cancer Center (Approval No. G2025-129-01) and conducted in accordance with the Declaration of Helsinki. Written informed consent was obtained from all participants.

### Design process

The research team consisted of breast surgery specialist nurses, rehabilitation therapists, clinicians, and industrial designers. The study followed a design thinking framework comprising five stages: Empathise, Define, Ideate, Prototype, and Test [15, 16].

#### Empathy and needs-analysis

Needs were explored through clinical observation, product and literature review, and semi-structured interviews. Clinical contextual observation was conducted in the ward, where the full process of nurse-led education and patient wall-climbing exercises was observed. Patients’ facial expressions, movement patterns, and common difficulties were recorded. Patients frequently experienced unstable finger support on smooth walls, compensatory movements due to insufficient grip, and variability in exercise execution. Nurses also demonstrated inconsistencies in instruction, with limited tools available for standardized demonstration and assessment. Existing wall-climbing rehabilitation devices and relevant literature were reviewed to analyse their functional characteristics and limitations. Most existing solutions lacked structured and visualized feedback on rehabilitation progress and were often bulky, difficult to install across settings, or limited in accessibility due to cost or procurement barriers. Semi-structured interviews were conducted at the study centre with twelve specialist nurses and ten postoperative breast cancer patients. The interview guide was informed by three feasibility domains: usability, adherence to training, and intervention effect. Patients reported discomfort during wall-climbing, difficulty maintaining home-based exercise, and challenges in perceiving functional improvement. Nurses highlighted difficulties in standardizing patient education, limited continuity of rehabilitation after discharge, and the lack of objective indicators for assessing recovery.

#### Define

Based on these findings, four key unmet needs were identified: lack of stable and comfortable finger support; absence of standardized and visualized progress feedback; bulky structure and complex installation limiting use across settings; and limited accessibility due to cost or procurement constraints. Accordingly, the core design challenge was defined as: “*How can we develop a standardized wall-climbing rehabilitation tool for postoperative breast cancer patients that is easy to install*,* affordable*,* and suitable for both hospital-based instruction and home use?*”

#### Ideate

The multidisciplinary team generated and evaluated multiple design concepts, including soft-hanging, adhesive-mounted, and rail-based structures. Concepts were screened based on safety, cost, ease of installation, and maintainability.

#### Prototype

A “vertical ladder with residue-free adhesive” concept was selected as the overall design approach. Multiple low-fidelity prototypes were developed to explore key structural parameters, including ladder length, width, groove spacing, and edge configuration. Nurses and postoperative breast cancer patients were invited to interact with the prototypes and provide feedback on tactile experience and comfort.

#### Test and optimization

**Fig. 1:**
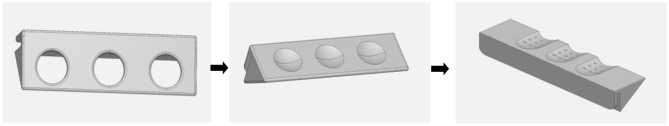
Iterative refinement of ladder surface design: perforated tructure→smooth surface→ microtextured surface with fine indentations

**Fig. 2:**
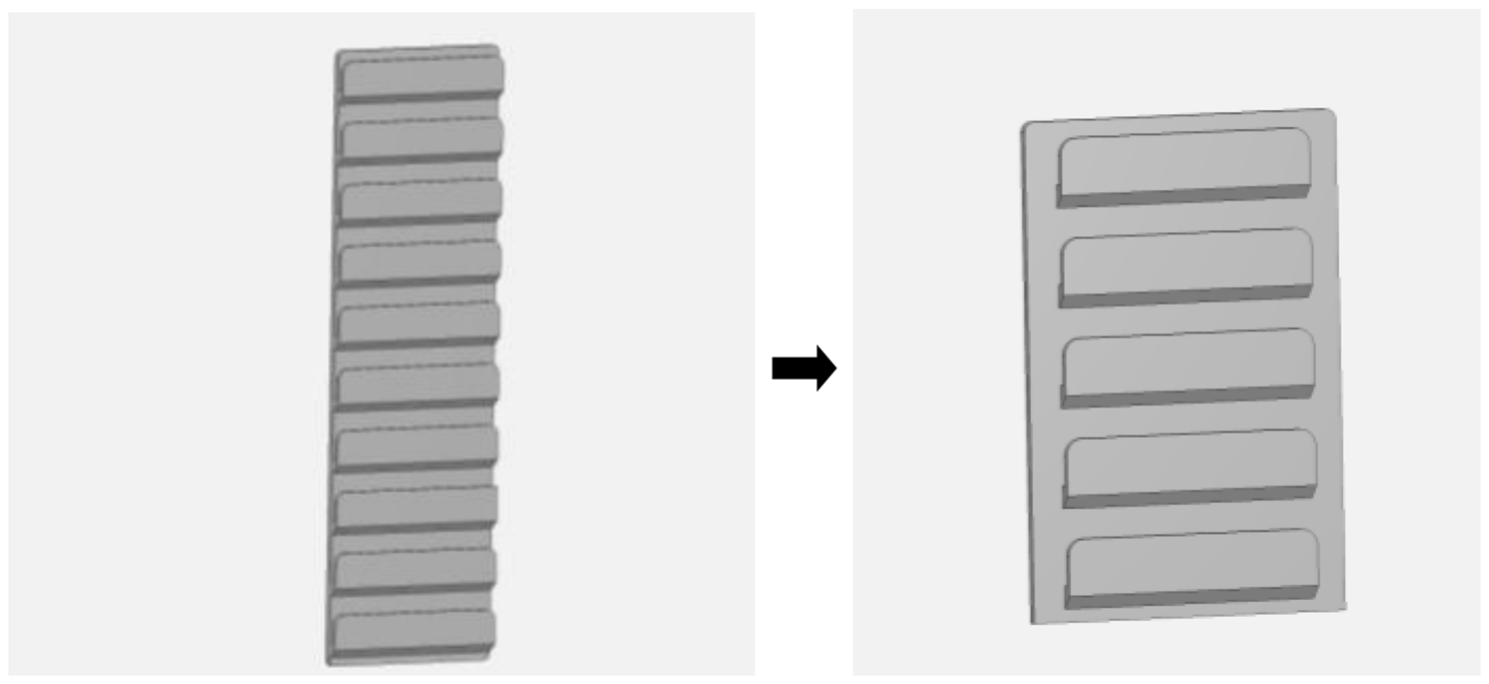
Iterative refinement of ladder height: embedded design (10 rungs per unit) → embedded design (5 rungs per unit)

**Fig. 3:**
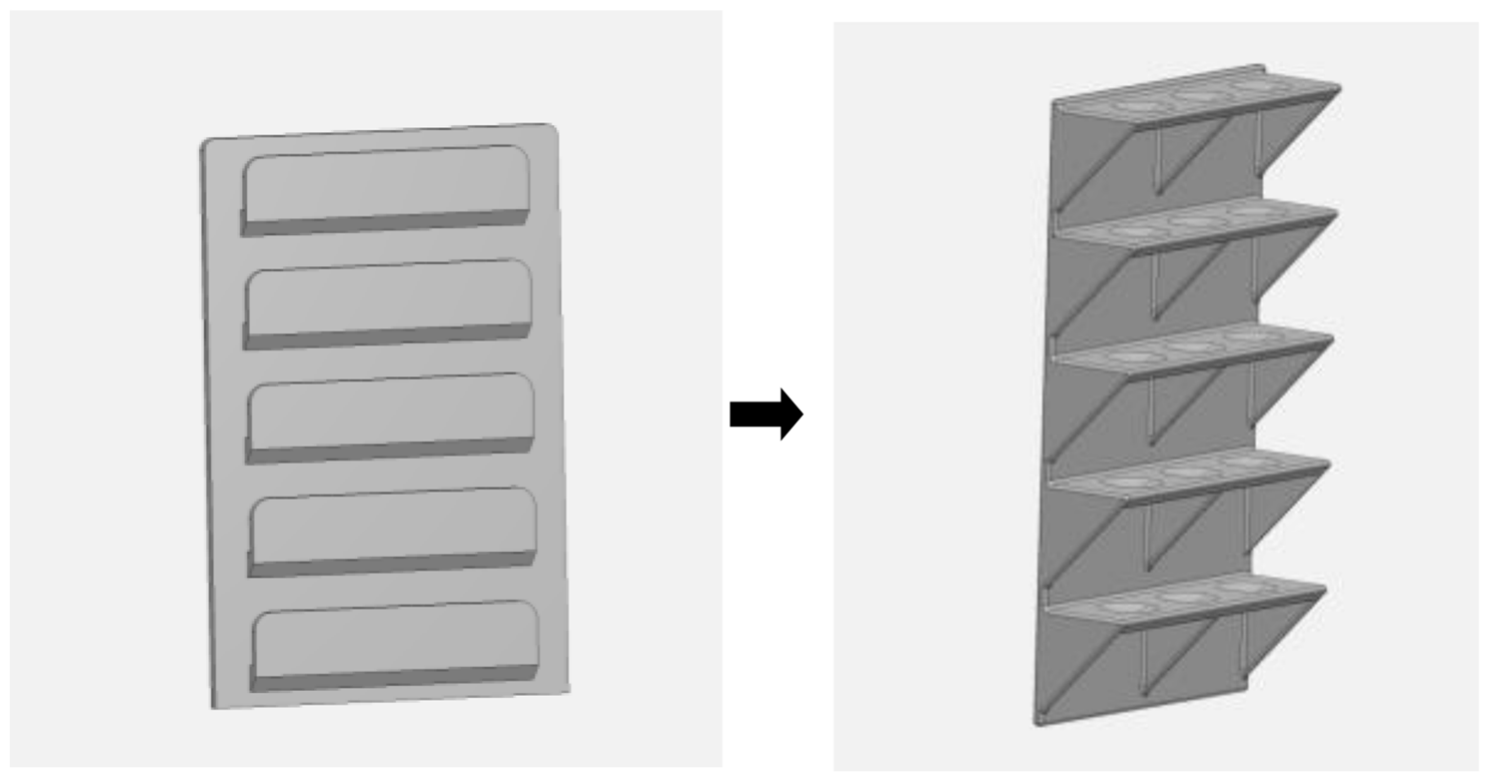
Evolution of ladder structure: embedded configuration → modular, non-embedded configuration

Iterative testing and user feedback informed progressive optimization of the design. Surface friction was refined from a perforated structure to a smooth surface and ultimately to a microtextured finish with fine indentations, balancing grip and finger mobility (Fig. 1). Unit height was reduced from 10 to 5 rungs to facilitate easier repositioning, improve height adjustability, and reduce material waste (Fig. 2). The structure was further evolved from an embedded to a modular, non-embedded design, enhancing installation and usability (Fig. 3). In addition, the size of the residue-free double-sided adhesive was increased to improve bonding across various wall surfaces, while edge rounding and surface finishing were optimised to enhance comfort and safety.

### Product structure and usage methodology

#### Structural composition and fundamental properties

**Fig. 4:**
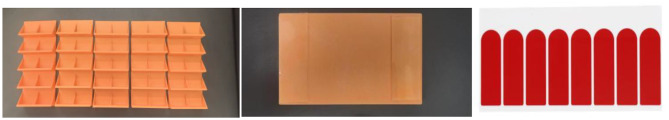
Structural composition and key components of the shoulder wall-climbing ladder, including the ladder units and adhesive tape system

The final shoulder rehabilitation wall-climbing ladder constitutes a non-medical rehabilitation aid, primarily comprising multiple longitudinal ladder units and residue-free double-sided adhesive tape: (1) Ladder Units: Presented as longitudinal strips, each unit features uniformly distributed, regular grooves along its front surface for sequential finger placement, functioning as physical “finger steps” and height graduations; Featuring rounded edges, the lightweight material boasts a smooth, easily cleanable surface. Each ladder unit measures 15 cm in height and 8 cm in width. Grooves are evenly distributed along the vertical axis on the front surface, with an inter-groove centre spacing of approximately 3 cm, enabling graded shoulder range-of-motion training for postoperative breast cancer patients of varying heights. The unit width and groove spacing were determined with reference to existing rehabilitation equipment parameters and refined through observational use, ensuring adequate finger support and operational comfort. The primary material is ABS engineering plastic, offering lightweight construction, high strength, wear resistance, and ease of cleaning. Combined with rounded chamfering, this ensures structural stability while enhancing tactile comfort and usage safety. (2) Residue-free double-sided adhesive tape: Used to secure the ladder to walls, suitable for various surfaces including emulsion-painted walls, tiles, smooth wooden panels, and glass. No drilling is required, and removal leaves no visible marks. (3) Modular assembly: Typically supplied with five ladder sections, which can be affixed sequentially from bottom to top. Patients and nursing staff can flexibly adjust the overall installation height according to height and bed elevation. See Fig. 4 for details.

#### Installation method

**Fig. 5:**
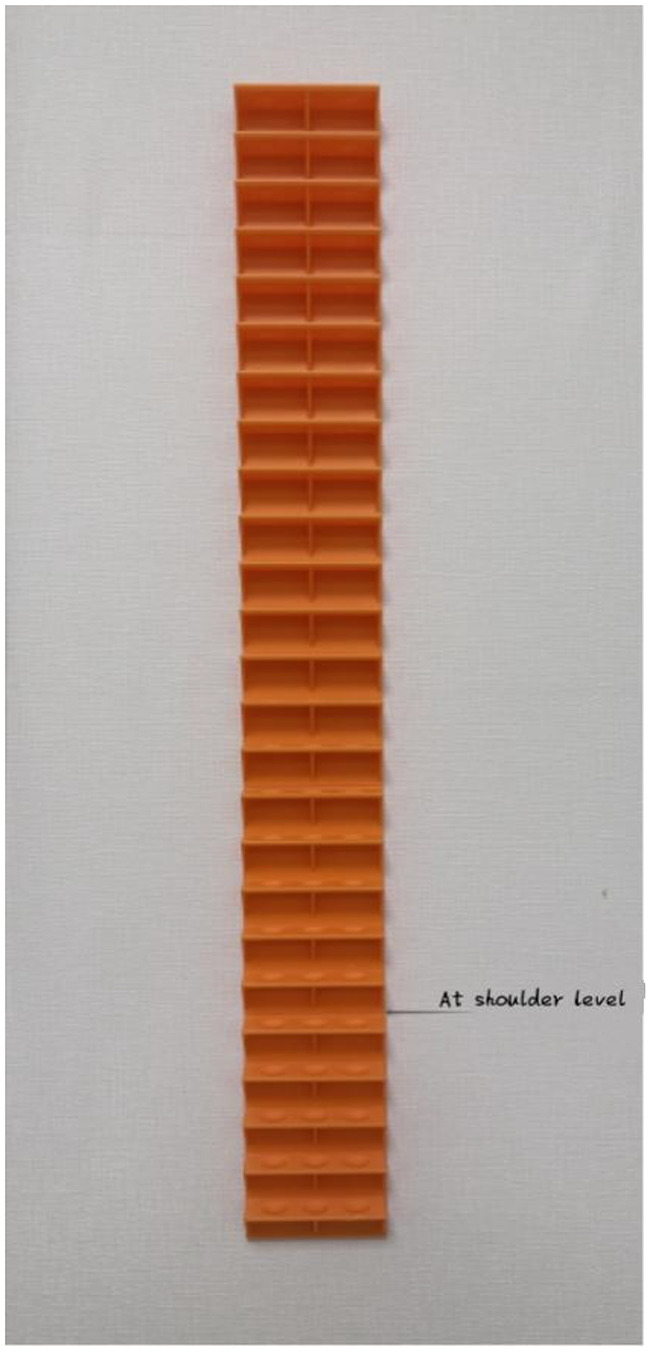
Installation method for shoulder wall-climbing ladder

Select a flat, stable, and unobstructed wall surface in the ward or home: (1) Estimate the appropriate height based on the patient’s stature; generally, position the top edge of the lowest ladder step level with the patient’s shoulder height; (2) Peel the protective layer from the residue-free double-sided adhesive tape and affix the ladder vertically to the wall from bottom to top, pressing firmly to secure. The adhesive tape (16 × 46 mm, thickness 0.5 mm) has a load-bearing capacity of up to 3.06 kg (30 N), which is approximately 43 times the weight of a single unit (71 g), ensuring a sufficient safety margin for secure wall mounting during routine use; (3) Inspect the connections between each ladder section for smooth alignment, ensuring no loose or curled edges. See Fig. 5 for details.

#### Standard usage procedure

**Fig. 6:**
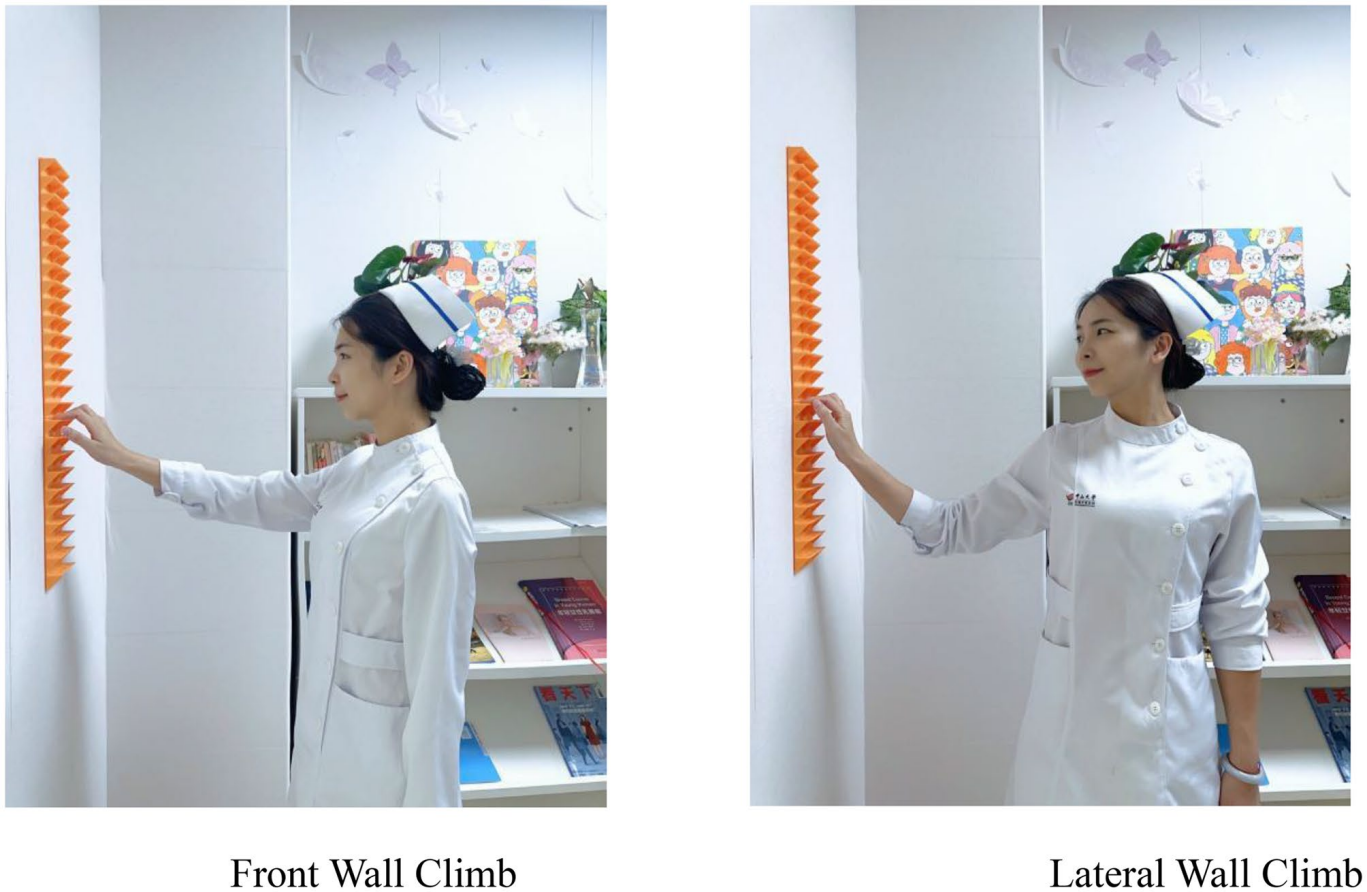
Standard movements for using the shoulder wall ladder. Note: Written informed consent for publication was obtained from the nurse depicted in the figure. The image contains no patient information

(1) Frontal wall climbing (shoulder joint forward flexion): The patient stands facing the wall ladder with feet shoulder-width apart, toes approximately 30 cm from the wall. Place the index and middle fingers of the affected hand in the lowest groove and progressively “climb” upwards along the grooves until a stretch sensation in the shoulder and tolerable pain are felt. Hold this position for five deep breaths, then slowly descend back to the starting position. (2) Lateral wall climb (shoulder abduction): The patient stands at a 90° angle to the wall, with the affected shoulder facing the ladder. The remaining steps are as above. See Fig. 6 for details.

### Application promotion model

#### Ward education scenario

**Fig. 7:**
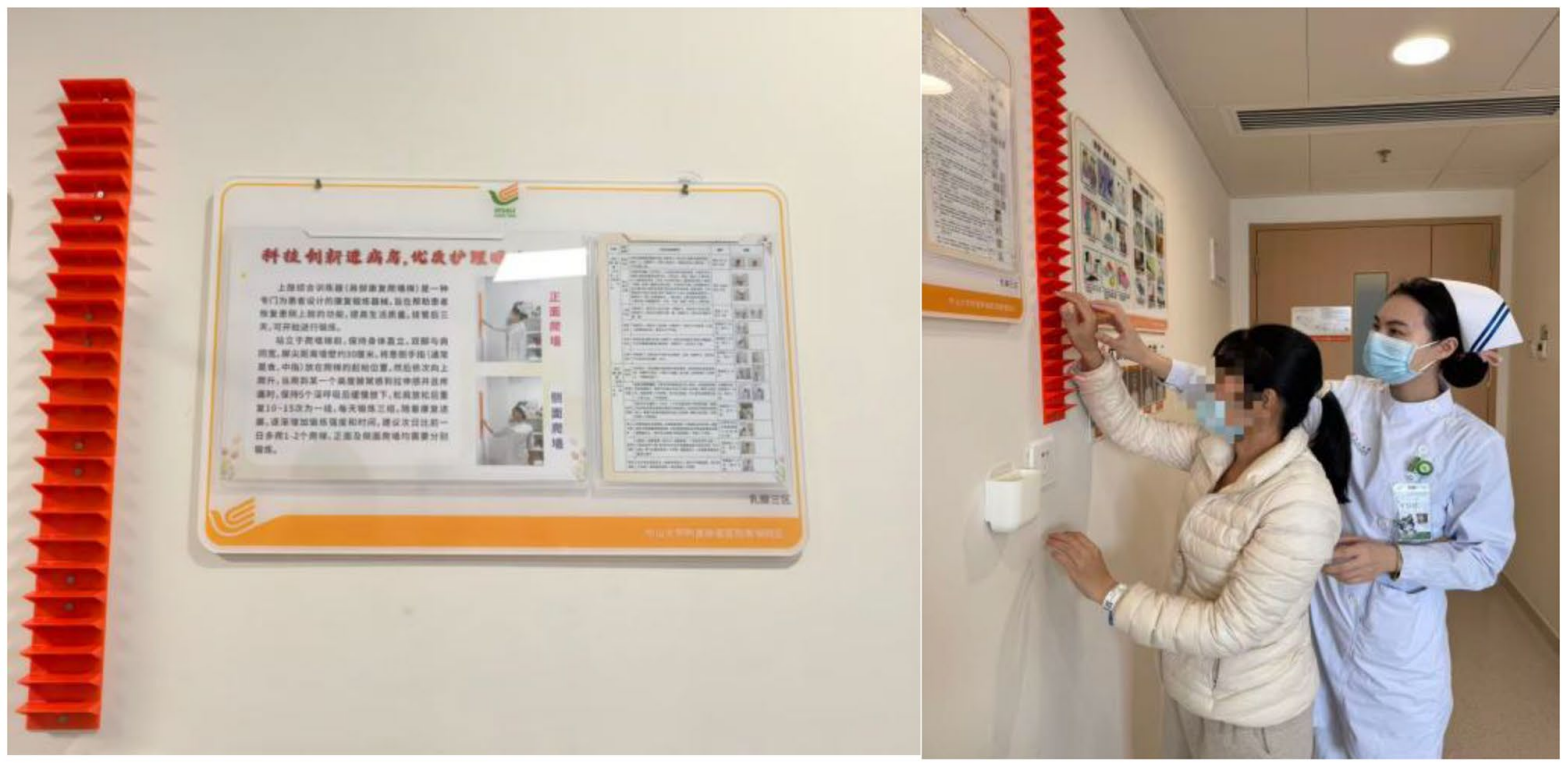
Ward education scenario. Note: Written informed consent for publication was obtained from the nurse; the patient has been anonymized by masking to preserve anonymity

Shoulder rehabilitation wall-climbing ladders are uniformly installed in breast surgery wards. During initial postoperative rehabilitation education, nurses demonstrate wall-climbing techniques in person and guide patients to practice daily after removal of chest wall and axillary drains during hospitalization. Patients are instructed to record the highest rung reached on the ladder that day as an interim goal. Post-discharge, patients follow exercise prescriptions for daily grouped practice, setting short-term progression targets of “climbing 1–2 additional notches” to tangibly track gradual improvements in shoulder joint mobility. See Fig. 7 for details.

#### Home rehabilitation setting

Prior to discharge, patients may purchase the same model of wall-climbing ladder through e-commerce platforms. Upon returning home, they can install and use it independently following the manual and nursing guidance, ensuring continuity between hospital and home training. The product has an average unit price of approximately ¥49 per item (approximately USD 7.0 or EUR 6.5), representing a low overall cost with sufficient gross margin to support future product iterations and channel maintenance. Patients may conveniently purchase the product through hospital-recommended channels or reputable e-commerce platforms such as Taobao, Xiaohongshu, and Douyin, facilitating a seamless extension of shoulder rehabilitation training into the home environment. This product has secured national design patent authorization, with Sun Yat-sen University Cancer Centre entering into an exclusive implementation license agreement with the manufacturing enterprise. The license is responsible for production and sales within China, providing institutional safeguards for the standardized transformation and long-term promotion of this nursing innovation.

#### Promotion and project implementation

Leveraging nationwide roadshows, academic conferences, and nursing professional societies, share post-mastectomy shoulder rehabilitation management expertise and the wall-climbing ladder application model to progressively enhance awareness and acceptance among similar departments. For healthcare institutions expressing interest in implementing the program, the enterprise or project team will provide one complimentary set of wall-climbing ladder products to their breast surgery/oncology department. This facilitates demonstration installations and patient education, supporting the establishment of specialised rehabilitation areas. Through standardized educational protocols, unified visual branding, and ongoing follow-up management, a replicable and scalable postoperative breast rehabilitation support program will be progressively established nationwide.

### Measurement instruments

#### Patient satisfaction questionnaire

Inclusion Criteria: ① Diagnosed with breast cancer and undergoing surgical treatment; ② Using the shoulder rehabilitation wall-climbing ladder for shoulder joint exercises for ≥ 2 weeks; ③ Possessing basic reading and comprehension abilities, and voluntarily participating in this study. As no validated, disease-specific instrument was available to assess user experience with this novel rehabilitation device, a self-developed questionnaire was used (see Appendix 1). The items were derived from clinical observations and patient interviews, focusing on key domains of feasibility and usability. The questionnaire comprised general demographic data and four domains: (1) Usability (Items 1, 2, 3, 5), including installation appropriateness, comfort, grip stability, and perceived safety; (2) Adherence to Training (Items 4, 6, 8), including visual progress tracking, home exercise continuity, and acceptability for long-term use; (3) Intervention Effect (Item 7), reflecting perceived improvement in shoulder range of motion and upper limb function; and (4) Overall Evaluation (Items 9, 10), including recommendation willingness and overall satisfaction. All 10 items were rated on a 5-point Likert scale (1 = strongly disagree to 5 = strongly agree; total score: 10–50), with higher scores indicating greater satisfaction. Satisfaction levels were categorized as high (≥ 40), moderate (30–39), or low (< 30). Two open-ended questions at the questionnaire’s conclusion collected patient experiences and improvement suggestions regarding the shoulder rehabilitation wall-climbing ladder. The questionnaire was distributed via the hospital’s follow-up system to patients who purchased the wall-climbing ladder at this institution.

#### Nurse experience questionnaire

Participants comprised breast surgery department nurses who had been using the wall-climbing ladder installed in their ward to guide patient exercises for ≥ 1 month. Similarly, the nurse experience questionnaire was self-developed to reflect clinical usage scenarios and practical considerations in nursing practice (Appendix 2). The questionnaire followed the same scoring format as the patient questionnaire (5-point Likert scale; total score: 10–50), with item content adapted to reflect a clinical perspective: (1) Usability (Items 1, 5, 8): ease of installation, enhancement of patient safety and comfort, and absence of additional nursing workload; (2) Adherence to Training (Items 3, 6, 7): ease of patient learning, in-hospital use willingness, and post-discharge exercise continuity; (3) Intervention Effect (Items 2, 4): facilitation of standardized rehabilitation education and assessment of rehabilitation progress; and (4) Overall Evaluation (Items 9, 10): perceived cost-effectiveness and scalability, and overall clinical endorsement. Two additional open-ended items invited nurses’ suggestions for improving clinical application. The questionnaire was anonymously administered via an online platform to eligible nurses in breast surgery departments of hospitals participating in the the LOVE BREAST program in which the wall-climbing ladder had been installed.

#### Statistical methods

Data analysis was conducted using SPSS statistical software. Continuous variables were presented as mean ± standard deviation (SD), and categorical variables were expressed as counts and percentages [n (%)]. Responses to the open-ended questions were collated and thematically summarized by the researchers.

## Results

### Design and development outcomes

Following multiple iterative cycles guided by the design thinking framework, the wall-climbing ladder underwent progressive refinement in surface texture, structural configuration, unit size, and adhesive performance based on user feedback at each stage. The final product successfully addressed the functional and usability needs identified during development and has been granted a Chinese national design patent (Patent No. ZL202430423041.6; Authorization Announcement No. CN309309737S).

### Scope of promotion and usage scale

The shoulder rehabilitation wall-climbing ladder has been progressively implemented across the breast surgery departments of 148 hospitals nationwide. In parallel, the device has been made commercially available through online e-commerce platforms, extending access to discharged patients and the broader public. From April to December 2025, cumulative sales exceeded 5,555 units, offering a practical and accessible home-based tool for upper limb functional rehabilitation among patients recovering from breast cancer surgery.

### Patient satisfaction outcomes

A total of 202 patient questionnaires were distributed, with 199 valid responses collected (response rate: 98.5%); the 3 invalid responses were excluded as the respondents had not undergone breast surgery. Demographic and clinical characteristics of patient respondents are presented in Table 1. The overall patient satisfaction score was 42.66 ± 5.30 points (out of 50), with domain scores and satisfaction level distribution detailed in Table 2. High satisfaction (≥ 40 points) was reported by 171 patients (86.0%), and only one patient reported low satisfaction (< 30 points). Open-ended responses indicated that patients primarily affirmed the wall-climbing ladder’s simplicity, ease of learning, convenience, and suitability for sustained home exercise. They noted that the stepped groove design facilitated leverage, enabled progressive range-of-motion enhancement, and provided visual rehabilitation progress tracking. A minority suggested incorporating strength training and traction-related functions.

**Table 1:** Demographic characteristics of patient respondents (*n* = 199)

Characteristic	*n* (%) / mean ± SD
**Age (years)**,** mean ± SD**	46.66 ± 9.22
**Gender**,** n (%)**	
Female	199 (100.0%)
Male	0 (0.0%)
**Education level**,** n (%)**	
Primary/secondary school or below	53 (26.6%)
College/diploma	81 (40.7%)
Bachelor’s degree or above	65 (32.7%)
**Breast surgery type**,** n (%)**	
Nipple-sparing mastectomy (NSM)	34 (17.1%)
Breast-conserving surgery	72 (36.2%)
Simple mastectomy	74 (37.2%)
Skin-sparing mastectomy (SSM)	19 (9.5%)
**Axillary management**,** n (%)**	
Sentinel lymph node biopsy	109 (54.8%)
Axillary lymph node dissection	90 (45.2%)
**Breast reconstruction**,** n (%)**	
Yes	34 (17.1%)
No	165 (82.9%)

**Table 2:** Patient satisfaction with the wall-climbing ladder

Domain	Score (mean ± SD)	Full score
Usability	4.26 ± 0.54	5
Adherence to Training	4.24 ± 0.56	5
Intervention Effect	4.29 ± 0.57	5
Overall Evaluation	4.23 ± 0.60	5
**Total Score**	**42.66 ± 5.30**	**50**
**Satisfaction level**,** n (%)**		
High satisfaction (≥ 40 points)	171 (86.0%)	—
Moderate satisfaction (30–39 points)	27 (13.5%)	—
Low satisfaction (< 30 points)	1 (0.5%)	—

### Findings on nursing staff usability

A total of 119 nurse questionnaires were distributed, with 119 valid responses returned (response rate: 100%). Demographic characteristics of nurse respondents are summarized in Table 3. The overall nurse experience score was 47.88 ± 3.49 points (out of 50), with domain scores and endorsement level distribution presented in Table 4. High endorsement (≥ 40 points) was reported by 115 nurses (96.6%), and no nurse reported low approval (< 30 points). Open-ended responses indicated that nurses generally perceived the wall-climbing ladder as simple to install, reasonably priced, and effective in standardizing and quantifying postoperative upper limb functional exercises, thereby promoting functional recovery and enhancing patient compliance. The device was also considered suitable for dissemination across multiple hospitals and e-commerce platforms. Suggestions focused on strengthening quantitative assessment and recording capabilities, and enriching educational materials such as illustrations and videos.

**Table 3:** Demographic characteristics of nurse respondents (*n* = 119)

Characteristic	*n* (%) / mean ± SD
**Age (years)**,** mean ± SD**	35.86 ± 6.3
**Gender**,** n (%)**	
Female	119 (100.0%)
Male	0 (0.0%)
**Education level**,** n (%)**	
Diploma	10 (8.4%)
Bachelor’s degree	101 (84.9%)
Master’s degree or above	8 (6.7%)
**Professional title**,** n (%)**	
Nurse	5 (4.2%)
Nurse-in-charge	29 (24.4%)
Senior nurse	85 (71.4%)
**Years in breast specialty nursing**,** mean ± SD**	11.36 ± 6.0
**Specialist training received**,** n (%)**	
Yes	102 (85.7%)
No	17 (14.3%)
**Duration of device use (months)**,** mean ± SD**	13.29 ± 6.0

**Table 4:** Nursing staff usability assessment of the wall-climbing ladder

Domain	Score (mean ± SD)	Full score
Usability	4.77 ± 0.41	5
Adherence to Training	4.74 ± 0.41	5
Intervention Effect	4.86 ± 0.34	5
Overall Evaluation	4.79 ± 0.37	5
**Total Score**	**47.88 ± 3.49**	**50**
**Endorsement level**,** n (%)**		
High endorsement (≥ 40 points)	115 (96.6%)	—
Moderate approval (30–39 points)	4 (3.4%)	—
Low approval (< 30 points)	0 (0%)	—

## Discussion

To our knowledge, this study represents the first application of design thinking to the development of a breast cancer rehabilitation product, and it has established a scalable dissemination pathway that has enhanced nurses’capacity for innovation. The wall-climbing ladder for shoulder rehabilitation, developed through design thinking, addresses genuine needs of breast cancer postoperative patients and clinical nurses.

### Design thinking addresses pain points of traditional wall-climbing training

Design thinking emphasizes empathy-driven problem identification, translating genuine clinical pain points into actionable design tasks through structured needs assessment [14]. In this study, this approach was applied to analyze limitations of existing rehabilitation modalities — including manual wall climbing, graduated stickers, and large fixed devices — with particular attention to comfort, standardization, and scalability in breast surgery ward and home rehabilitation contexts. Key design challenges were identified, including stable upper-limb support, visualized progress tracking, dual-setting adaptability, and cost-installation feasibility. A multidisciplinary team then conducted iterative development through brainstorming and low-fidelity prototyping, with successive refinements targeting groove morphology, ladder dimensions, spacing, and material selection. This user-centered “trial–revise–retry” cycle reflects the core prototyping principles of design thinking [15]. Compared with conventional top-down development, this approach may facilitate earlier identification of impractical design features and support preliminary feasibility assessment, though formal quantitative comparisons were not conducted.

### Advantages of integrating ward education with home use

Conventional shoulder rehabilitation devices are typically confined to hospital settings, creating a discontinuity in training conditions after discharge [12]. The present product was developed around a “ward-to-home” concept: identical ladders installed in hospital wards allow nurses to deliver standardized instruction during early postoperative recovery, potentially helping patients establish consistent movement patterns prior to discharge. Upon returning home, patients may access the same product through hospital-recommended or commercial channels. When installed under comparable conditions, this may help reduce environmental discrepancies between inpatient and home settings, potentially supporting rehabilitation continuity and adherence. While multi-center adoption and user feedback indicate good acceptability, the clinical effectiveness of this approach requires confirmation through controlled trials with standardized outcome measures.

### Pathways for translating nursing innovations and prospects for dissemination

Despite a growing number of nursing innovation patents, effective translation pathways remain underdeveloped. This case outlines one such pathway, encompassing clinical problem identification, iterative prototyping, patent application, departmental piloting, multi-center implementation, and online dissemination. Nursing staff functioned not only as users and educators but as initiators and co-developers throughout this process. This case suggests that frontline clinical experience may facilitate meaningful collaboration between nursing teams, designers, and industry partners, offering a potentially transferable framework whose generalizability warrants further validation across diverse settings and product types.

### Limitations and outlook

This study retains several limitations: First, evaluation primarily relied on subjective user feedback and a self-developed questionnaire, without standardized objective outcome measures or fully validated instruments. Moreover, although multi-center adoption and product utilization suggest feasibility, key claims regarding comfort, standardization, and scalability remain insufficiently supported by systematic quantitative evidence. Future studies should employ validated assessment tools and controlled multi-center designs, incorporating objective indicators such as functional outcomes, adherence rates, and comfort scales. Second, the current product focuses mainly on range-of-motion training and lacks integration with digital health technologies. Future development could incorporate mobile applications and smart systems to enable automated data collection, remote monitoring, and personalized rehabilitation programs.

## Conclusion

This study illustrates a feasible nursing innovation pathway that integrates design thinking with multi-centre implementation and real-world dissemination. The developed device appears good usability across ward and home settings and may support standardised rehabilitation and continuity of care after breast cancer surgery. Further controlled studies are warranted to evaluate clinical effectiveness and long-term outcomes.

## Electronic supplementary material

Below is the link to the electronic supplementary material.

Supplementary Material 1

Supplementary Material 2

## Data Availability

The datasets generated and analyzed during the current study are available from the corresponding author on reasonable request. For data sharing beyond this, please contact the corresponding author.

## Abbreviations

SPSS: Statistical Package for the Social Sciences
