# Supplementary material for: Research on the design and innovative transformation path of shoulder rehabilitation wall-climbing ladders for postoperative breast cancer patients based on design thinking
Source: BMC Nurs. 2026 Apr 27;25:535. doi: 10.1186/s12912-026-04689-7 (PMC13261912; doi:10.1186/s12912-026-04689-7)
Supplement: Supplementary file 1 — Supplementary Material 1 [file 12912_2026_4689_MOESM1_ESM.docx]

# **Appendix 1: Patient Satisfaction Questionnaire for the Shoulder Rehabilitation Wall-Climbing Ladder**

****Instructions:****
This questionnaire aims to assess your experience with the Shoulder Rehabilitation Wall-Climbing Ladder, in order to help us improve the product and patient education. The questionnaire is anonymous, and all information will be used for research purposes only. Please respond based on your actual experience.

### **Section I: General Information**

****Gender:**** □ Male □ Female

****Age:**** ______ years

****Education level:****
□ Primary school or below □ Junior high school □ Senior high school / Vocational school □ Associate degree □ Bachelor's degree or above

****Type of breast surgery:****
□ Simple mastectomy □ Breast-conserving surgery □ Nipple-sparing mastectomy (NSM) □ Skin-sparing mastectomy (SSM)

****Axillary management:****
□ Sentinel lymph node biopsy (SLNB) □ Axillary lymph node dissection (ALND)

****Breast reconstruction:**** □ No □ Yes — Type:
□ Immediate implant-based reconstruction □ Immediate autologous tissue reconstruction □ Delayed reconstruction □ Other: ________

****Time since surgery:**** ______ months

****Affected side:**** □ Left □ Right

****Is the surgical side your dominant hand (e.g., used for writing or eating)?****
□ No □ Yes

****Did you receive upper limb exercise education during hospitalisation?****
□ Yes □ No

### **Section II: Experience and Satisfaction Scale**

****Scoring instructions:****
1 = Strongly disagree; 2 = Disagree; 3 = Neutral; 4 = Agree; 5 = Strongly agree.
Please select one number for each item.

| **No.** | **Item** | **Score** |
| --- | --- | --- |
| 1 | The installation position and height of the wall-climbing ladder are appropriate for my exercise needs. | 1　2　3　4　5 |
| 2 | Using the wall-climbing ladder for "finger wall-climbing" is more comfortable than climbing on a plain smooth wall. | 1　2　3　4　5 |
| 3 | The grooves on the wall-climbing ladder provide "finger steps" that help me gain leverage and maintain stability. | 1　2　3　4　5 |
| 4 | By observing which rung I can reach, I can clearly track my rehabilitation progress. | 1　2　3　4　5 |
| 5 | I feel safe and confident when exercising with the wall-climbing ladder. | 1　2　3　4　5 |
| 6 | Using the wall-climbing ladder helps me continue exercising at home following the method taught in hospital. | 1　2　3　4　5 |
| 7 | I feel that using the wall-climbing ladder has improved the range of motion of my affected shoulder and upper limb function. | 1　2　3　4　5 |
| 8 | The appearance and colour of the wall-climbing ladder are suitable for long-term home use and do not make me feel uncomfortable or self-conscious. | 1　2　3　4　5 |
| 9 | I would recommend this wall-climbing ladder to other patients who have undergone breast cancer surgery. | 1　2　3　4　5 |
| 10 | Overall, I am satisfied with my experience of using the Shoulder Rehabilitation Wall-Climbing Ladder. | 1　2　3　4　5 |

****Scoring:****
Total score range: 10–50 points. Higher scores indicate greater patient satisfaction.
Categorisation: ≥40 points = High satisfaction; 30–39 points = Moderate satisfaction; <30 points = Low satisfaction.

### **Section III: Open-Ended Questions (optional)**

1. What do you like most about this wall-climbing ladder?

______________________________________________________

1. What aspects do you think need improvement?

______________________________________________________
