# Supplementary material for: Research on the design and innovative transformation path of shoulder rehabilitation wall-climbing ladders for postoperative breast cancer patients based on design thinking
Source: BMC Nurs. 2026 Apr 27;25:535. doi: 10.1186/s12912-026-04689-7 (PMC13261912; doi:10.1186/s12912-026-04689-7)
Supplement: Supplementary file 2 — Supplementary Material 2 [file 12912_2026_4689_MOESM2_ESM.docx]

# **Appendix 2: Nurse Experience Questionnaire for the Shoulder Rehabilitation Wall-Climbing Ladder**

****Instructions:****
This questionnaire is designed to capture your real-world experience of using the Shoulder Rehabilitation Wall-Climbing Ladder in clinical practice to guide postoperative rehabilitation exercises for breast cancer patients. The questionnaire is anonymous and for research purposes only. Please respond based on your actual clinical experience.

### **Section I: General Information**

****Gender:**** □ Male □ Female

****Age:**** ______ years

****Highest educational qualification:****
□ Associate degree or below □ Bachelor's degree □ Master's degree or above

****Professional title:****
□ Nurse □ Senior Nurse □ Charge Nurse or above

****Years of experience in breast surgery nursing:**** ______ years

****Have you received specialist training in postoperative functional exercise for breast cancer patients?****
□ Yes □ No

****Duration of using the wall-climbing ladder to guide patient exercises:**** ______ months

### **Section II: Experience Scale**

****Scoring instructions:****
1 = Strongly disagree; 2 = Disagree; 3 = Neutral; 4 = Agree; 5 = Strongly agree.

| **No.** | **Item** | **Score** |
| --- | --- | --- |
| 1 | The installation and removal of the wall-climbing ladder are straightforward and do not significantly increase workload. | 1　2　3　4　5 |
| 2 | Having the wall-climbing ladder uniformly installed in the ward facilitates standardised postoperative rehabilitation education. | 1　2　3　4　5 |
| 3 | When demonstrating the "finger wall-climbing" technique using the ladder, patients find it easier to understand and perform the correct posture. | 1　2　3　4　5 |
| 4 | Observing which rung a patient can reach helps me assess the range of motion of the affected shoulder and monitor rehabilitation progress. | 1　2　3　4　5 |
| 5 | Compared with climbing on a plain smooth wall, the wall-climbing ladder enhances patients' sense of safety and comfort during exercise. | 1　2　3　4　5 |
| 6 | Patients are generally willing to try and use the wall-climbing ladder for training during hospitalisation. | 1　2　3　4　5 |
| 7 | Patients who continue using the same wall-climbing ladder at home after discharge (e.g., via e-commerce platforms) are likely to demonstrate improved continuity and adherence to home-based exercises. | 1　2　3　4　5 |
| 8 | Incorporating the wall-climbing ladder into routine nursing practice does not noticeably increase my workload. | 1　2　3　4　5 |
| 9 | In terms of price and accessibility, I consider this product to have good cost-effectiveness and potential for wider dissemination. | 1　2　3　4　5 |
| 10 | Overall, I hold a positive view of the wall-climbing ladder's application in postoperative functional rehabilitation for breast cancer patients, and I am willing to continue using it and recommend it to other departments. | 1　2　3　4　5 |

****Scoring:****
Total score range: 10–50 points. Higher scores indicate greater clinical endorsement.
Categorisation: ≥40 points = High endorsement; 30–39 points = Moderate endorsement; <30 points = Low endorsement.

### **Section III: Open-Ended Questions (optional)**

1. In your view, what is the greatest advantage of the wall-climbing ladder in clinical application?

______________________________________________________

1. What improvements would you suggest for this product or the related patient education process?

______________________________________________________
